# Analogue experiments to investigate magma mixing within dykes

**DOI:** 10.1007/s00445-025-01809-0

**Published:** 2025-03-28

**Authors:** Tegan A. Havard, Thomas J. Jones, Janine L. Kavanagh

**Affiliations:** 1 Department of Earth, Ocean and Ecological Sciences, University of Liverpool, Liverpool, L69 3GP UK; 2 Lancaster Environment Centre, Lancaster University, Lancaster, LA1 4YQ UK

**Keywords:** Magma mixing, Magma mingling, Dyke, Volcanic plumbing system, Analogue experiment

## Abstract

Multiple magmas residing in plumbing systems that feed fissure eruptions can physically and chemically interact and mix during storage, transport, and eruption. The extent and success of such mixing ultimately control the physical properties (e.g. density and viscosity) of the magma, the eruptive conditions, and thus the associated hazards. Analogue experimental studies have previously investigated magma interactions in plumbing systems typically with pipe-like or chamber-like geometries (i.e. cylindrical or cuboidal respectively) and immiscible fluids that represent magma mingling. However, these findings are difficult to extrapolate to high aspect ratio geometries typical of dykes that characterise fissure systems. Here, we present results from a high aspect ratio experimental setup to explore magma mixing within dykes. Using an array of miscible fluid pairs, representing magmas of differing composition, we found that flow is initially localised towards the centre of the system and mixing occurs at the interface between the two fluids, spreading laterally out over time. The mixing interface is generally greater, and mixing is more rapid when the starting physical properties of the two fluids are more similar. Furthermore, a dyke-like geometry facilitates mixing to a greater degree relative to a chamber-like system. We explore the implications of the mixing dynamics on diffusive and crystal exchange between magmas, the transport of magmas through the crust, and the evolution of physical and chemical properties of interacting magmas. The mixing ratio trends of our experimental data are similar to near-real time geochemical mixing data from the Kīlauea 2018 eruption, suggesting a future avenue for understanding the complexities of mixing during magma ascent.

**Supplementary Information:**

The online version contains supplementary material available at 10.1007/s00445-025-01809-0.

## Introduction

Different magmas can physically and chemically interact within a volcanic plumbing system, both during storage within deep magma reservoirs and during transport as magma ascends to the surface through dykes (e.g. Gansecki et al. 2019; Gibson and Walker 1963). This mingling and mixing can change the physical and chemical properties of the bulk magma, affecting the type of eruptive products and eruptive behaviour over time (e.g. Gansecki et al. 2019), and can potentially trigger an eruption (e.g. Sparks et al. 1977). Basaltic eruptions are often fed by dykes and can display a wide range of eruptive behaviour, from effusive to explosive (e.g. Carracedo et al. 2022; Gansecki et al. 2019; Jones et al. 2022; Pedersen et al. 2022) fundamentally controlled by the fluid dynamics and transport of the magma within the subsurface (e.g. Cassidy et al. 2018; Gonnermann and Manga 2007; Houghton et al. 2004). Understanding the dynamics of subsurface magma transport and the role of magma mixing is therefore important to better manage the evolving hazard associated with these fissure systems (e.g. Houghton et al. 1999).

Distinct magmas can interact through mixing and mingling. These terms, mixing and mingling, are often used interchangeably in the literature, and here, we use the definitions selected in Jarvis et al. (2021), whereby mixing is defined as the chemical interaction between two or more magmas that produce intermediate compositions, and mingling is the physical interaction between multiple magmas. Magma mingling is generally captured in the macroscopic and outcrop scale, recorded as heterogeneous material with distinct boundaries between the different composition magmas. Examples include mafic enclaves (e.g. Barbarin and Didier 1992; Caricchi et al. 2012), composite dykes and sills (e.g. Askew et al. 2014; Buist 1959; Ubide et al. 2014), and banded pumice (e.g. Perugini and Poli 2012). Whereas, evidence for magma mixing tends to be recorded at the microscale and in the geochemistry. This evidence ranges from zoning of phenocrysts (e.g. Landi et al. 2004; Sakuyama 1984), resorption textures recorded in crystals (e.g. Castro et al. 1990), chemically distinct crystal populations (e.g. Holness et al. 2015), rounded or embayed crystals (e.g. Craig and Fodor 2018), and evolving geochemical trends in eruptive products (Gansecki et al. 2019; Halldórsson et al. 2022) to fully hybridised, homogeneous products (e.g. Humphreys et al. 2010). Both processes, mixing and mingling, frequently occur together with mixing creating a more diffuse boundary between the mingled products (e.g. Jarvis et al. 2021).

The inability to directly observe what occurs within active subsurface plumbing systems means that we rely on indirect methods to interpret dynamic processes (e.g. Eibl et al. 2017; Geiger et al. 2016). Analogue experiments are one example of an indirect method. They allow us to investigate a simplified natural system on laboratory time and length scales, and when scaled correctly, the results are applicable to the natural system of interest (Merle 2015). Previous analogue experimental studies aimed to investigate the mixing/mingling of magmas in plumbing systems have typically used cuboidal, chamber-like geometries (Cardoso and Woods 1996; Turner and Campbell 1986; Woods and Cowan 2009) or cylindrical, conduit-like geometries (Beckett et al. 2011; Blake and Campbell 1986; Debacq et al. 2003; Huppert and Hallworth 2007; Koyaguchi 1985; Koyaguchi and Blake 1989; Palma et al. 2011; Séon et al. 2005) and often used immiscible fluids that represent physical magma mingling rather than chemical mixing. They show that an initially stratified magma source can interact and mingle during ascent within a conduit geometry, the extent of mingling may be limited within a chamber-like geometry, and that flow regimes, mixing and mingling extent, and qualitative flow behaviours are dependent on viscosities and viscosity contrasts between the experimental fluids. More recently, high aspect ratio slot geometries—a more applicable geometry for dykes and fissures—have been used with immiscible fluids to investigate flow processes such as localisation in fissure systems (Capponi et al. 2020; Cole et al. 2023; Jones and Llewellin 2021; Pioli et al. 2017). Jones and Llewellin (2021) investigate convective exchange flow using immiscible fluids and show that a more diverse range in flow behaviour is observed than that of cylinders, and flow becomes focussed within the analogue dyke, explaining flow focussing along an eruptive fissure (e.g. localisation during the 2022 Meradalir eruption; Krmíček et al. 2022).

Here, we present a scaled analogue experimental study to investigate interactions of miscible fluids in a dyke-like geometry. We describe the scaling of the apparatus and present the evolution of flow patterns, interface length, and the spatial and temporal change in mixing. We discuss the results in the context of the 2018 Kīlauea eruption, which is a natural basaltic fissure system with evidence of mixing, to illustrate how these laboratory results could be applied to natural eruptions.

## Methods

### Experimental procedure

**Fig. 1:**
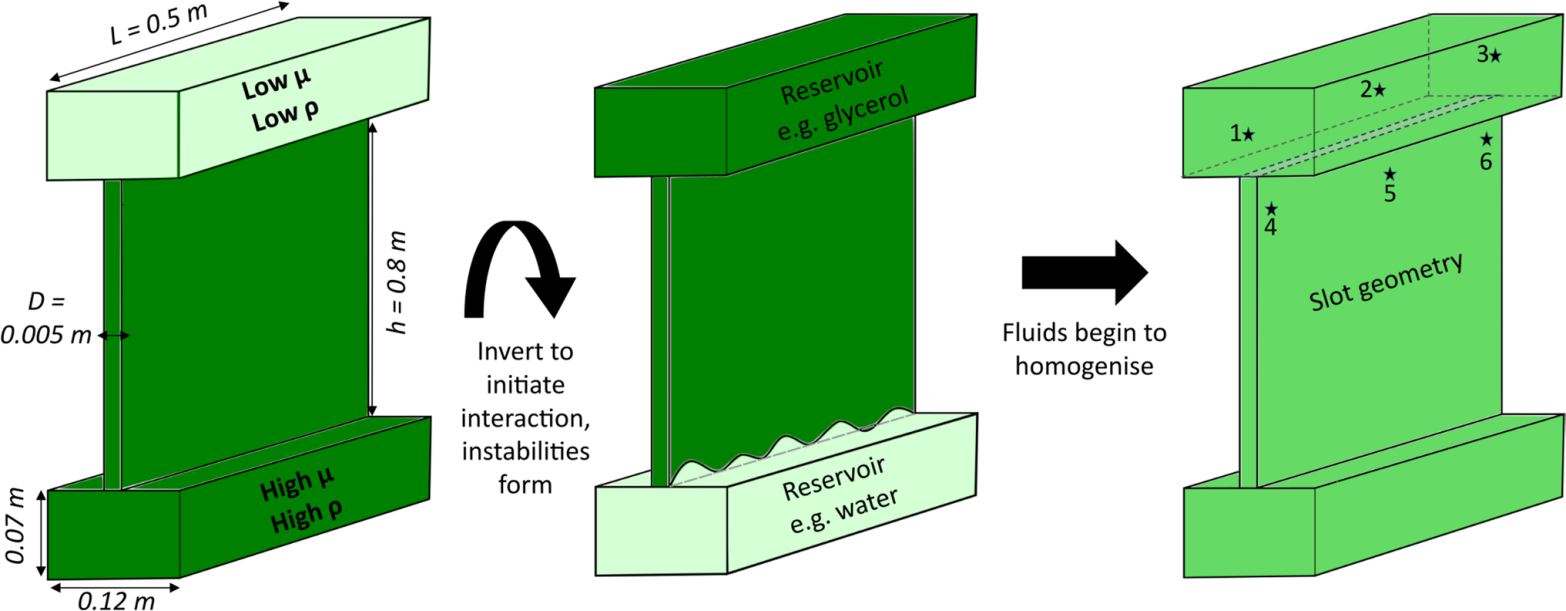
Sketch of the experiment setup and brief methodology. The clear Perspex tank is initially filled to the top of the slot with the higher-density fluid (shown in dark green) followed by the lower-density fluid (shown in pale green). The tank is then inverted to begin fluid interaction and images captured throughout the experiment. At the end of an experiment, samples were collected from six locations indicated by the stars, and the remaining fluid in the tank was collected and homogenised

The analogue experiments were carried out in an I-beam shape tank (Fig. 1) made with 2-cm-thick transparent acrylic panels. The tank comprised a slot of dimensions 0.5 × 0.8 × 0.005 m (Fig. 1; length, *L* × height, *h* × thickness, *D*) with two reservoirs of dimensions 0.5 × 0.07 × 0.12 m (*L* × *h* × *D*). A frame freely suspended the 10.4 litre capacity tank above the ground to allow 360° rotation around the horizontal axis. The tank design allowed observation of flow dynamics with minimal to no deflection of panels (design based on Jones and Llewellin (2021)). The slot region represents the dyke geometry of interest, and the reservoirs are not representative of a natural feature but allow the experiment to run for longer durations. All filming and subsequent image analysis was focused on the central slot (i.e. analogue dyke). The setup was indirectly illuminated from behind.

Two different density miscible fluids were used in each experiment. Sugar-based dye (Wilton® violet gel icing colour; typically, 0.5 g/l) was added to one of the fluids if the fluid colours were too similar to be later separated using image analysis techniques. The apparatus was initially filled to the top of the slot by the relatively dense fluid (or within 15 cm of the slot top under circumstances of finite fluid supply). The fluid was left for 1 to 3 h to allow the bubbles to settle out; then, the fluid temperature was measured, and the lower-density fluid was slowly added on top until the tank was full. The temperature of the second fluid was then recorded, and lastly, the system was sealed closed and airtight. Samples of the starting fluids were retained for later analysis of their physical properties.

Once the fluid preparation and experiment preparation were complete, the stopwatch was started and the tank was immediately inverted to start the experiment, minimising the time for any pre-mixing of the miscible fluids to occur. A Canon EOS 1200D DSLR camera with 18–55 mm zoom lens recorded experiment images at up to 3 frames per second using the continuous shooting mode, with longer manually triggered frame intervals up to one frame per hour used for experiments that displayed slower interactions. The camera setup consisted of a f/4.5 aperture, 1/100 shutter speed, ISO 3200, focal length of 23–24 mm, and the camera was positioned viewing the slot plane 1.26 m away from the tank and 0.71 m above the ground. At the end of the experiment (the time where the bottom reservoir is exhausted of upwelling material or after > 24 h if there is still upwelling material), six fluid samples were collected from the tank using a syringe at the locations indicated in Fig. 1: three were taken from the upper section of the slot, and three from the upper reservoir. All fluid in the tank was then drained, collected, and homogenised by mixing the fluids with a paddle mixer in an external container to produce a fully mixed bulk end fluid that was sampled. The physical properties (density and viscosity) of all the samples were measured at the recorded temperatures and a common reference temperature of 24 °C (see section on ‘Physical characterisation of fluids’).

### Physical characterisation of fluids

Miscible Newtonian fluids with differing viscosities and densities were selected as magma analogues in this study. These included water (tap water), glycerol (Gly; vegetable glycerine supplied by Lubrisolve), diluted glycerol (dGly), diluted golden syrup (dGS), golden syrup (GS; Lyle’s Golden Syrup), and golden syrup mixed with glycerol (glyGS). We used seven unique miscible fluid pairs: water and dGly, water and Gly, water and glyGS, water and GS, dGly and Gly, Gly and dGS, and Gly and GS. Dilutions (using tap water) and mixtures were prepared by mixing the fluids together with a paddle mixer for 5 min and left overnight to allow any bubbles introduced during fluid preparation to rise out. The same process was applied for adding dye to a fluid. Generally, 10 l of the high-density fluid and 5 l of the low-density fluid were prepared per experiment.

The densities of the fluid samples were measured using 5, 10, or 25 ml calibrated glass pycnometer flasks (dependent on the sample size available). All samples were run at two temperatures: (1) at the temperature recorded when the sample was collected and (2) at a common reference temperature of 24 °C. The pycnometer flask was filled with the fluid using a syringe, bubbles were left to settle out if present, the flask placed in a water bath, and left to equilibrate to the required temperature for 15 min. Once the sample was at temperature, the flask was removed, dried, and its mass measured using a mass balance (± 0.001 g). The density was calculated by dividing the fluid mass by the known, calibrated pycnometer volume. To calculate a representative measurement error, the most viscous fluid (golden syrup) was measured seven times in the smallest pycnometer flask (5 ml). This combination was chosen as it represents the largest possible error sources; the pure golden syrup was the most difficult to fill the pycnometer flask and the small pycnometer produces the greatest relative volume error. The error on the fluid density was calculated using the standard deviation as a percentage of the mean and then applied to all other density measurements as a maximum uncertainty (± 0.6%).

To characterise the viscosity of the fluid samples, rotational rheometry was used. This was conducted on a Haake MARS III rotational rheometer, using a cone plate geometry (60 mm diameter and 1° slope cone angle). Approximately 1 ml of sample was added to the Peltier temperature control base plate, the upper cone lowered to leave a gap of 52 microns between the cone and base plate, and the sample was left to thermally equilibrate for 2 min. The rotational tests were carried out at controlled shear rates between 0.001 and 1000 s^−1^ over 60 measurements, with 10 shear stress measurements per decade distributed logarithmically. Viscosity is calculated from shear stress within the RheoWin software. Due to experimental limitations, data collected at low shear rates can be unreliable dependent on the fluid viscosity (Ewoldt et al. 2015). Therefore, we calculated the viscosity by averaging the viscosity data over a different shear rate range depending on the viscosity: 100–1000 s^−1^ for fluid viscosities up to 0.001 Pa s, 10–1000 s^−1^ for viscosities up to 0.1 Pa s, 1–1000 s^−1^ for viscosities up to 10 Pa s, and 0.1–1000 s^−1^ for viscosities greater than 10 Pa s. To quantify a representative error of the measurements, the viscosity of the fluid that showed the most variability (pure glycerol) was measured five times and the percentage error of the mean applied to all other viscosity measurements as a maximum uncertainty (± 6%).

### Image measurement methods

#### Ascent velocity and interface shape

In the initial stages of the experiments, instabilities developed along the interface between the two fluids. To quantify the interface length between the fluid phases at a common stage (i.e. when the first growing lobe reached a given height in the tank), images captured during the experiment were imported into Fiji, the image processing package (Schindelin et al. 2012, version 1.54f). The contact between the fluids was manually traced with a line 1-pixel thick; thus, the number of pixels measured is equivalent to the length in pixels. The distance was converted into centimetres using the known dimensions of the tank visible in the picture (50 cm length). A typical cropped image size was ~ 2000 × ~ 3000 pixels giving a resolution on the order of ~ 40 pixels/cm.

The velocity of the fluid ascending in the slot was determined by measuring the tip velocity of ascending fluid lobes. The lobe that first reached the top reservoir was chosen and tracked back through the photos to its initial appearance. The distance between the lobe front and the top reservoir was determined using the Measure function in Fiji, with the image scale set using known dimensions of the tank. The time taken for the lobe to travel the distance was calculated using the stopwatch time in the image, or if unavailable, from the image metadata.

#### Image processing—quantifying mixing

The extent and efficiency of mixing between the two fluids were obtained through quantifying the change in pixel values in Matlab. An independent calibration of how pixel value relates to mixing was achieved by capturing images of the slot filled with different dilutions of dyed glycerol, showing that the pixel value varies linearly with dye concentration and fluid density (Fig. S1 in Online Resource 1). This allows the pixel value to be used as a proxy for mixing across the slot area for the experiment duration.

Before working with the mixing image data, three stages of pre-processing and calibration were completed for each experiment dataset. Firstly, in stage one, to remove the effect of uneven light illumination across the slot, a photo of the slot filled with the dense fluid was taken (if there was insufficient quantity of this fluid, the empty tank was used). The image was processed by converting the image to greyscale and then to double precision (i.e. a number format that allows calculations to be performed on the matrices). Three iterations of anisotropic diffusion were applied to reduce noise and the mean pixel value of the slot area calculated. Then, a matrix was created to calculate the difference between the mean pixel value and the measured value to provide a background map of pixel intensity across the imaged plane. This pixel intensity map was subtracted from all future images after the anisotropic diffusion stage to correct for the uneven illumination and quantify the relative colour change.

Secondly, in stage two, to calibrate the images by colour, the slot was filled with one endmember experiment fluid, an image captured, and stage one pre-processing up to calculating mean pixel value was applied. The average pixel value of the slot was measured. This was repeated for the second end member fluid. These calibrations provided the upper and lower limits for pixel values in an experiment.

Lastly, in stage three, to allow comparison between experiments, the pixel values in images were normalised and plotted between 0 (the lower limiting pixel value, the denser fluid) and 1 (the upper limiting pixel value, the less dense fluid; Fig. S2 in Online Resource 1). The evolving fluid properties could then be visualised as the pixel value is directly related to the density and viscosity of the fluid (Fig. S1). Here, we focus on mapping density changes within the slot as there is a linear relationship between fluid density and the pixel value, making the later analysis simpler (viscosity has an exponential relationship). All processing scripts are available in an accompanying data publication (Havard et al. 2025).

#### Image processing and analysis—quantifying fluid evolution

To quantify how mixing evolved over time as the fluids interacted during flow through the slot, mixing at a common height of ~ 4 cm below the slot top was measured. This height was chosen as it was near the top of the slot but far enough away to mitigate against potential edge effects from the top fluid reservoir. To quantify how mixed the fluids were, we normalised any measured value at the chosen height, *x*_*i*_, to the ideal fully mixed fluid value of the whole tank, *x*_*m*_. The variable *x* can represent densities, geochemical values, or as in the case for image processing, the pixel values. A mixing ratio, *β*_*i*_ was then calculated, where 1 indicates the fluid is fully mixed, and 0 that the fluid is completely unmixed.

1 $${\beta }_{ia}=\frac{{x}_{i}-{x}_{\text{min}}}{{x}_{m}-{x}_{\text{min}}}$$

2 $${\beta }_{ib}=\frac{{x}_{i}-{x}_{\text{max}}}{{x}_{m}-{x}_{\text{max}}}$$

where *x*_min_ is the minimum possible value, and *x*_max_ is the maximum possible value. When *x*_*i*_ ≤ *x*_*m*_, Eq. (1) is used; for cases where *x*_*i*_ ≥ *x*_*m*_, Eq. (2) is used.

We constructed a sequence of the normalised pixel row data from multiple images to visualise the spatial arrangement of mixed and unmixed material near the top of the slot, how mixed the products were and how mixing evolved over time. Furthermore, we took the sum of this normalised data row, *β*_sum_ for every image to see the changes in the bulk material over time. Because we normalise between unmixed (0) and mixed (1), a greater *β*_sum_ means that the material reaching the selected height is more mixed.

The image timestamp was converted to dimensionless time, t^*^, to enable comparison across experiments using:

3 $${t}^{*}=\frac{{t}_{i}-{t}_{0}}{{t}_{\text{end}}-{t}_{0}}$$

where *t*_*i*_ is the time of interest, *t*_0_ is the initial time, and *t*_end_ is the final time. *t*_end_ is defined as the time where the bottom reservoir is exhausted of upwelling material.

Two experiments had upwelling material remaining after > 12 hours and were stopped prematurely, and for a third experiment, the time gap between the final ten images was too large to determine whether there was upwelling material remaining. Therefore, for these three experiments, t^*^ could not be calculated and the experiment data are included here solely for qualitative data visualisation purposes.

### Dimensional analysis

Analogue experiments need to be scaled to the natural system in order for the results to be meaningful (Galland et al. 2018; Jones and Ehlers 2021; Kavanagh et al. 2018; Merle 2015). The behaviour of natural and experimental systems can be linked by the creation of a set of dimensionless quantities. Here, we use the Buckingham Pi Theorem (Buckingham 1914) method to identify the relevant dimensionless quantities, or groups, important to understanding dynamics of magma interaction within dyke-fed fissure systems.

The Reynolds number, Re is the ratio of inertial and viscous forces during fluid flow:

4 $$\text{Re}=\frac{{\rho }_{d}\mathit{{UR}}}{{\mu }_{d}}$$

where *ρ*_*d*_ is the density of the downwelling fluid, *U* is the velocity of ascending fluid, *R* is the representative length scale, which in our case is the thickness of the slot, *D*, and *µ*_*d*_ is the viscosity of the downwelling fluid.

The density ratio, ρ^*^:

5 $${\rho }^{*} =\frac{{\rho }_{u}}{{\rho }_{d}}$$

where *ρ*_*u*_ is the density of the upwelling fluid.

The viscosity ratio, μ^*^:

6 $${\mu }^{*}=\frac{{\mu }_{u}}{{\mu }_{d}}$$

where *µ*_*u*_ is the viscosity of the upwelling fluid.

The aspect ratio, γ which is expressed as:

7 $$\upgamma =\frac{D}{L}$$

where *L* is the length of the opening.

The experiments were designed, and the measured quantities are such, that there is overlap in the range of natural values for basaltic dykes and experimental values for these dimensionless parameters (Table 1). This means that these experiments show dynamic similarity with natural systems and can be used to give insight into magma mixing within basaltic dyke-like geometries.

**Table 1:** The parameter and dimensionless number values for the experiments and natural basaltic dykes. Natural values are obtained from the literature cited and references therein, and experimental values are measured during this study. † indicates values calculated from variables given here

Parameters and dimensionless numbers (Units)		Experiment	Nature	Example references
*Parameters*				
*ρ*_*u*_	Upwelling fluid density (kg m^−3^)	997–1260 (water, glycerol)	900 (bubble rich basalt)–2600	Jones and Llewellin (2021)
*ρ*_*d*_	Downwelling fluid density (kg m^−3^)	1160–1450 (diluted glycerol, golden syrup)	2600–2900	Lesher and Spera (2015)
*Δρ*	Density difference (kg m^−3^)	1–450	1–2000	†
*L*	Dyke/slot length (m)	0.5	10^2^–10^5^	Gonnermann and Taisne (2015)
*D*	Dyke/slot thickness (m)	0.005	1–10	Gonnermann and Taisne (2015)
*µ*_*u*_	Upwelling fluid viscosity, (Pa s)	9.4 × 10^−4^–1	10^1^–10^4^	Lesher and Spera (2015)
*µ*_*d*_	Downwelling fluid viscosity (Pa s)	1.2 × 10^−2^–61	10^1^–10^4^	Lesher and Spera (2015)
*U*	Velocity (m s^−1^)	7 × 10^−5^–0.12 (upwelling lobe velocity)	10^−2^–10^1^ (dyke propagation velocity)	Rutherford et al. (2000)
*g*	Gravity (m s^−2^)	9.81	9.81	
*Dimensionless numbers*				
Re	Reynolds number	2 × 10^−5^–60	10^−4^–10^2^	Glazner (2014)
ρ*	Density ratio	0.69–0.99	0.40–0.85	†
μ*	Viscosity ratio	2 × 10^−5^–18	10^−3^–10^0^	†
*γ*	Aspect ratio	10^–2^	10^−3^–10^−4^	†

## Results and analysis

Seven experiments are described in this study, each with a unique miscible fluid pair (Table 2). First, a qualitative description of the flow evolution for the experiments is given, followed by interface length measurements, and the spatial and temporal evolution of mixing in the slot. All results are grouped by the upwelling fluid material (water, followed by diluted glycerol, and then glycerol), and within the groups, they are arranged in order of increasing downwelling fluid density.

**Table 2:** Table of experimental parameters, results, and dimensionless groups for each experiment. Fluid abbreviations are glycerol = Gly and golden syrup = GS. Fluid dilutions are with water apart from Experiment W3 where the downwelling fluid is golden syrup diluted with glycerol. *Subscript u*, upwelling fluid property; *Subscript d*, downwelling fluid property; *ρ*, density; *ρ*_*b*_, bulk density (measured from the homogenised total fluid volume of the tank at the experiment end); *µ*, viscosity; *V*, starting volume (total tank volume is 10,400 cm^3^); *t*_end_, time at which fluid is no longer upwelling (or when indicated by †, the time from the last image used of a prematurely ended experiment); *U*, velocity (measured from the advancing front of the lobe that reaches the top reservoir first). The following dimensionless groups are calculated using measured values. ρ*, density ratio; µ*, viscosity ratio; Re, Reynolds number

Experiment name	Upwelling fluid	Downwelling fluid	*ρ*_*u*_	*ρ*_*d*_	*ρ*_*b*_	*μ*_*u*_	*μ*_*d*_	*V*_*u*_	*t*_*end*_	*U*	ρ*	μ*	Re
			kg m^-3^	kg m^-3^	kg m^-3^	Pa s	Pa s	cm^3^	s	m s^-1^	-	-	-
W1_W-dGly	100 wt% Water	61 wt% Gly	999	1159	1093	9.3 x 10^-4^	1.2 x 10^-2^	4450	116	1.2 x 10^-1^	8.6 x 10-^1^	7.6 x 10^-2^	5.7 x 10^1^
W2_W-Gly	100 wt% Water	100 wt% Gly	997	1253	1167	9.4 x 10^-4^	4.2 x 10^-1^	4200	615	2.6 x 10^-2^	8.0 x 10^-1^	2.2 x 10^-3^	3.8 x 10^-1^
W3_W-GlyGS	100 wt% Water	42 wt% Gly, 58 wt% GS	998	1371	1228	9.8 x 10^-4^	5.1 x 10^0^	4200	2202	5.5 x 10^-3^	7.3 x 10^-1^	1.9 x 10^-4^	7.4 x 10^-3^
W4_W-GS	100 wt% Water	100 wt% GS	999	1440	1268	1.0 x 10^-3^	4.0 x 10^1^	4200	6459	1.0 x 10^-3^	6.9 x 10^-1^	2.6 x 10^-5^	1.9 x 10^-4^
D1_dGly-Gly	99 wt% Gly	100 wt% Gly	1255	1257	1256	6.7 x 10^-1^	8.3 x 10^-1^	4200	†22181	6.9 x 10^-5^	1.0 x 10^0^	8.1 x 10^-1^	5.2 x 10^-4^
G1_Gly-dGS	100 wt% Gly	64 wt% GS	1259	1276	1272	7.6 x 10^-1^	4.1 x 10^-2^	4500	†15172	5.8 x 10^-3^	9.9 x 10^-1^	1.8 x 10^1^	8.9 x 10^-1^
G2_Gly-GS	100 wt% Gly	100 wt% GS	1259	1452	1371	1.0 x 10^0^	6.1 x 10^1^	4200	†25896	1.8 x 10^-4^	8.7 x 10^-1^	1.7 x 10^-2^	2.2 x 10^-5^

### Qualitative description of the flow evolution

**Fig. 2:**
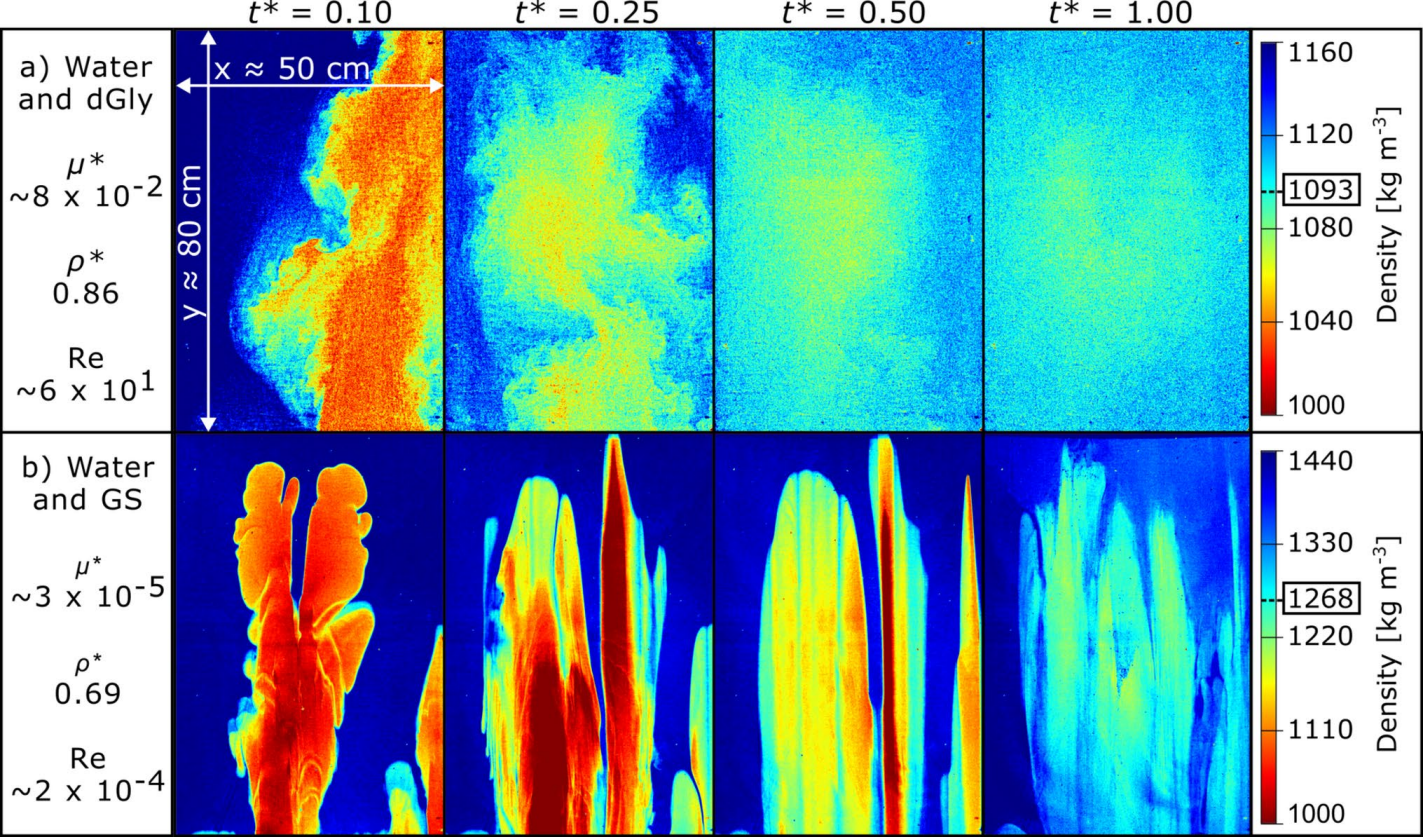
Examples of processed images from **a** water-dGly and **b** water-GS showing the morphology of lobes and variation in density at common dimensionless times, t*. Colour bar ranges from red (*ρ*_*u*_, less dense upwelling fluid) to blue (*ρ*_*d*_, denser downwelling fluid). Bulk density, *ρ*_*b*_, is identified by the box around the number. The Reynolds number, Re, viscosity and density ratios, µ* and ρ*, respectively, are shown for each fluid pair. Scale for all images ≈ 50 × 80 cm (*L* × *h*)

Across all experiments, instabilities developed at the interface between the fluids once the tank had been inverted. These are recognised by the formation of a series of lobes that appear on the order of seconds to minutes, dependent on the fluid combination used (Video Fig. S1 in Online Resource 2). Some of the individual lobes grew and ascended, whilst others stagnated or began to decay/drain. We interpret these to be Rayleigh–Taylor instabilities (Rayleigh 1882) based on their shape and the origin of their formation (i.e. high-density fluid above lower-density fluid), but they are not the focus of this study. Most of the experiments showed little mixing initially, with interfaces remaining sharp, clearly separating lobes of less dense fluid within a background of denser fluid. Through time, mixing occurred at the interface between fluid pairs in all experiments, with the interface becoming more diffuse over time (Fig. 2). Once an upwelling lobe was established in the centre of the slot, new lobes travelled upwards and expanded laterally and, if close enough, coalesced into adjacent lobes towards the slot centre (Video Fig. S1).

**Fig. 3:**
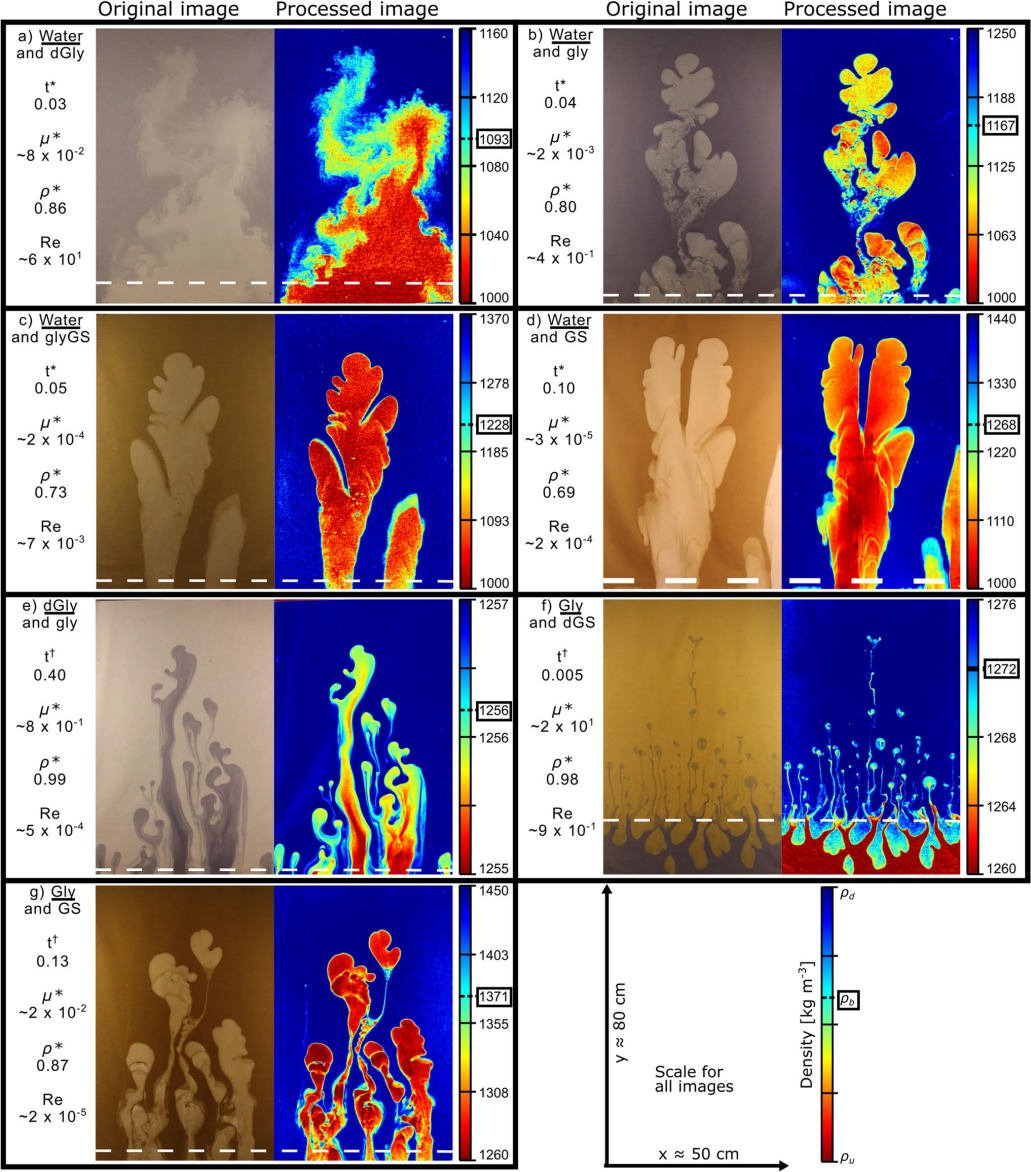
The original and processed images showing early stages of fluid interaction in each experiment once the first lobe reached a height of 70 cm. The density, based on the pixel value, is shown by the colour bar (red = *ρ*_*u*_ = less dense upwelling fluid, blue = *ρ*_*d*_ = denser downwelling fluid). Bulk density, *ρ*_*b*_, is identified by the box around the number. The initial interface between the two starting fluids is shown by the white dashed line. The Reynolds number, Re, dimensionless time, t* (*t*^*†*^ for experiments that ended prematurely), and viscosity and density ratios, µ* and ρ*, respectively, are shown for each fluid pair, and the upwelling fluid name is underlined. **a** = water-dGly (±0.021, i.e. the processed image pixel value error when pixel values are between 0 and 1); **b** = water-Gly (± 0.014); **c** = water-glyGS (± 0.019); **d** = water-GS (± 0.009); **e** = dGly-gly (± 0.010); **f** = gly-dGS (± 0.017); **g** = gly-GS (± 0.012). Scale for all images ≈ 50 × 80 cm (*L* × *h*)

For experiments using water as the initial upwelling fluid, typically one or two lobes were sustained (Fig. 3). The interface between water and diluted glycerol (dGly) quickly became diffuse, rapidly incorporating denser fluid into an upwelling plume in a turbulent manner (Fig. 3a; Video Fig. S1a). Water upwelling through glycerol (Gly; Fig. 3b; Video Fig. S1b), glycerol-golden syrup mixture (glyGS; Fig. 3c; Video Fig. S1c), or golden syrup (GS; Fig. 3d; Video Fig. S1d) showed similar features with the advancing lobe front splitting into multi-lobed heads.

When diluted glycerol or pure glycerol was the upwelling fluid (Fig. 3), many more lobes were sustained, relative to the water experiments. Up to seven lobes were sustained as dGly upwelled through Gly, with bifurcation of the lobe fronts (Fig. 3e; Video Fig. S1e). For experiments with upwelling Gly, tens of narrow (0.1–1 cm) lobes were formed with diluted golden syrup (dGS) as the downwelling fluid; the lobe fronts repeatedly disconnected from the glycerol fluid reservoir to form approximately 1 cm diameter elliptical to spherical blobs, travelling ahead of the main body of upwelling fluid (Fig. 3f; Video Fig. S1f). Gly upwelling in GS produced four growing lobes that were sustained (i.e. lobes remained connected to the lower reservoir) with the lobe fronts bifurcating (Fig. 3g; Video Fig. S1g).

### Mixing interface

**Fig. 4:**
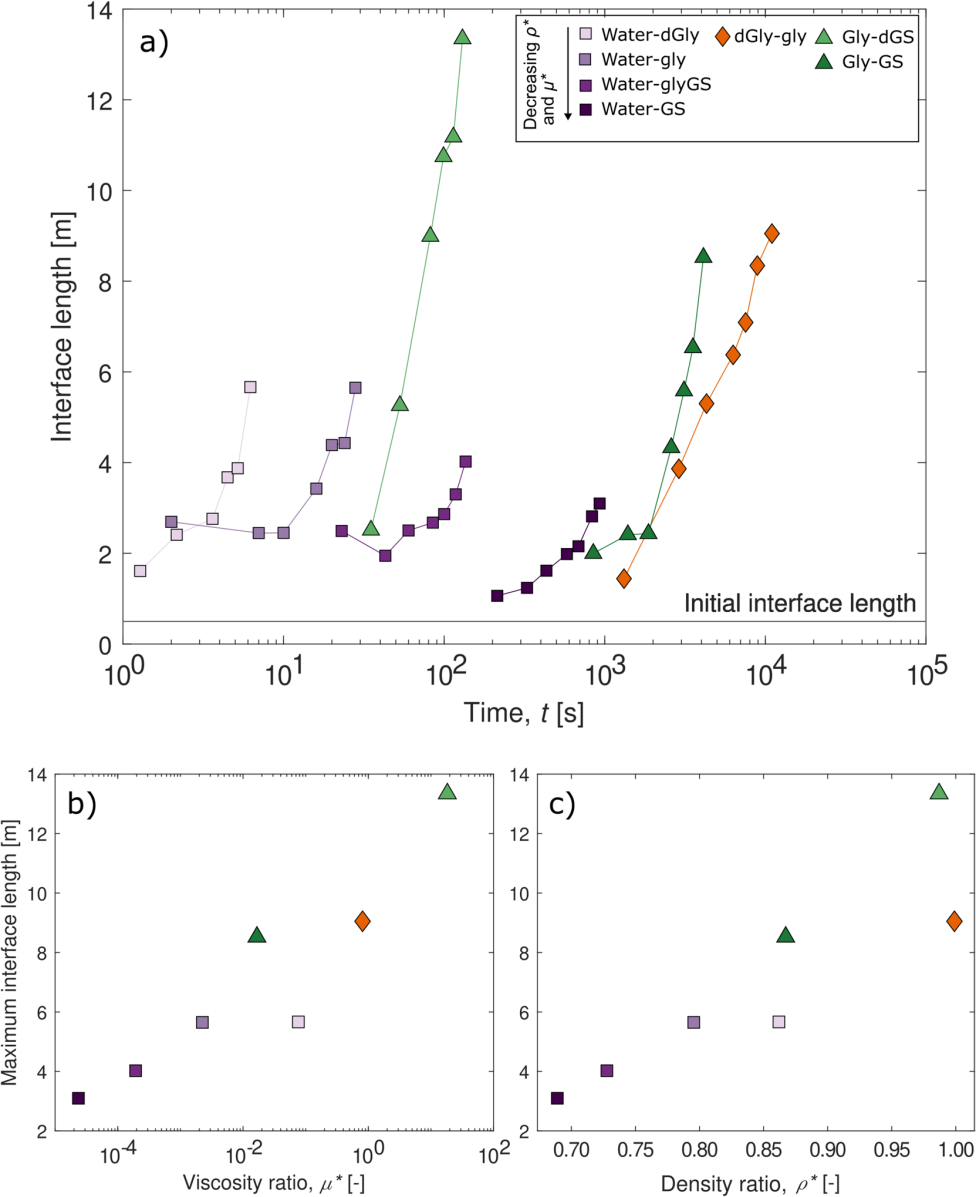
**a** Plot of manually traced interface length as the first ascending lobe reached common heights in the slot (every 10 cm) as a function of time. **b** Maximum interface length as the first ascending lobe reached the top of the slot as a function of viscosity ratio, µ*, and **c** as a function of density ratio, ρ*. Symbols are the same for all plots: upwelling water experiments = square, light purple to dark purple for decreasing ρ* and µ*; upwelling diluted glycerol experiment = orange diamond; upwelling glycerol experiments = triangle, light green to dark green for decreasing ρ* and µ*

The length of the interface between the dense downwelling fluid and the less dense upwelling fluid was measured as a function of time until upwelling material entered the top reservoir (Fig. 4a). This shows that the experiments with the same upwelling fluid, when compared with each other, require longer durations to grow the interface when the viscosity ratio and density ratio of the fluid combination were lower (Fig. 4a; Table 2). Experiments with upwelling dGly and Gly produced the longest interface lengths, matching the qualitative observation that they sustained the greater number of growing lobes (Fig. 3). The water-Gly and water-GlyGS experiments show a dip in the interface length during the early stages of lobe growth. This is likely due to the stagnation and decay of lobes at the expense of the single growth of one main lobe (Video Figs. S1b and S1c). The maximum interface length, the length of the interface as the first ascending lobe reached the top of the slot, also varies as a function of the density and viscosity ratio (Fig. 4b and c). For experiments with water as the upwelling fluid, the maximum interface length generally increases with increasing density ratio and for increasing viscosity ratio. For upwelling glycerol experiments, the maximum interface length also increases with increasing viscosity and density, although more data is needed to confirm the trend.

### Evolution of mixing

**Fig. 5:**
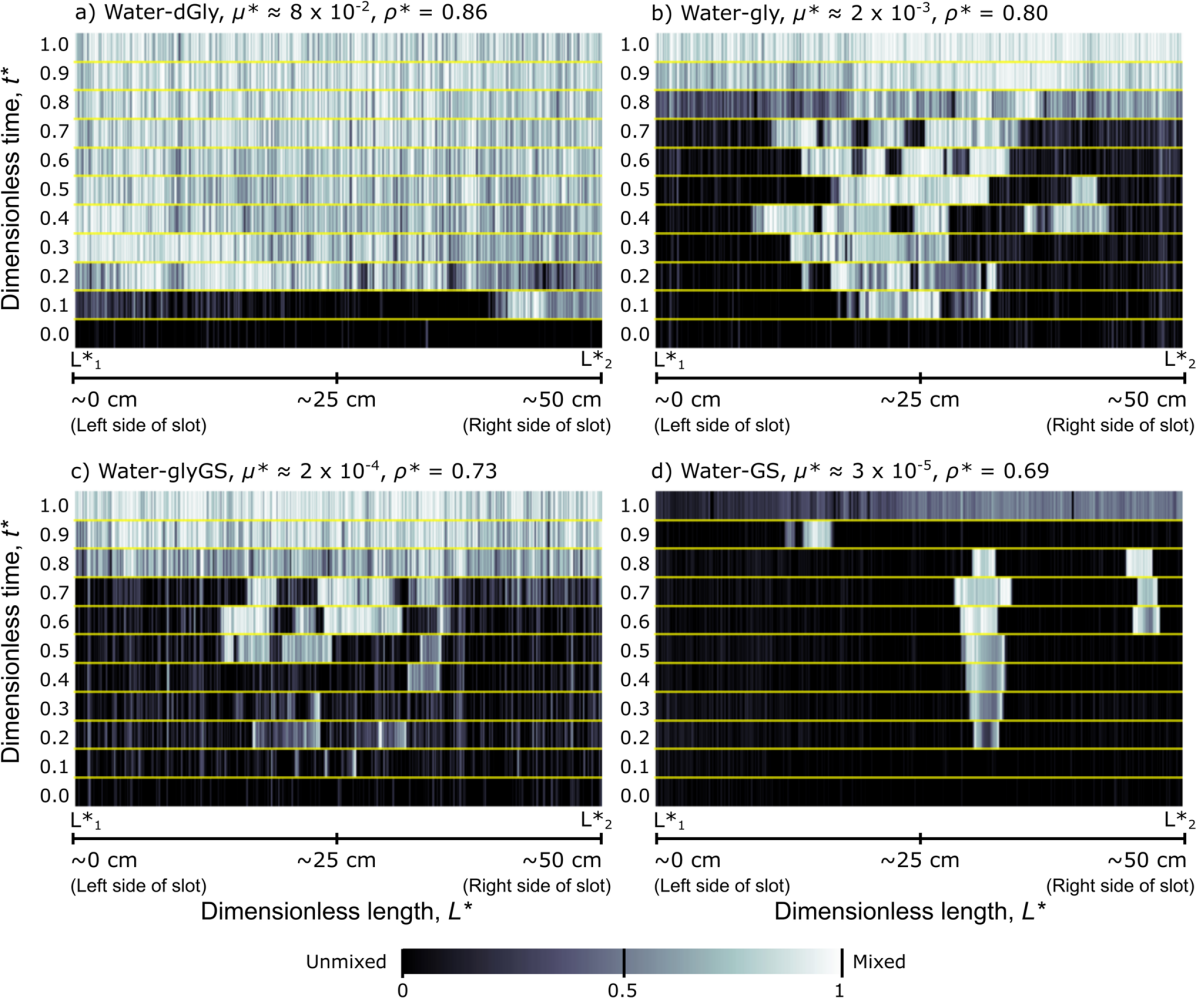
Spatiotemporal mixing sequences showing how mixed the material is across the length of the slot (at a height of ~ 76 cm up from the slot base) and how this changes over time. The *x*-axis is the dimensionless length of the slot (L*_1_–L*_2_; approximately 50 cm), and the *y*-axis is dimensionless time, t*. The dark end of the spectrum represents unmixed material (0), and the light represents more mixed (1). The yellow lines separate each timepoint in a sequence. The viscosity and density ratios, µ* and ρ*, respectively, are shown for each fluid pair. **a** = water-dGly; **b** = water-Gly; **c** = water-glyGS; **d** = water-GS

The dynamics of mixing within the slot changes both spatially and temporally. To illustrate this, we construct spatiotemporal diagrams, where the material within a given length across the slot at a height of ~ 76 cm (from the slot base) is captured at common dimensionless time points for experiments where the bottom reservoir was completely exhausted (Fig. 5). The evolution of material mixing is now described here for the water experiments by increasing density of the second fluid.

For water-dGly (Fig. 5a), the zone of mixing is narrow (~ 10 cm) for only a short amount of time before the zone widens and occupies the full length (50 cm) of the slot by t* = 0.3 and remains relatively consistent for the rest of the experiment. For water-Gly (Fig. 5b), water-glyGS (Fig. 5c), and water-GS (Fig. 5d), the mixing zone occupies a narrow section (~ 3–15 cm) towards the centre of the slot for a longer period of the experiment duration. There are more discrete patches of mixed material in water-Gly than water-glyGS, and the same in turn for water-GS. This is most likely linked to the amount of lobe bifurcation occurring, which more readily occurred during the water-Gly experiment. For water-Gly (Fig. 5b) and water-glyGS (Fig. 5c), mixed material is more dispersed across the length of the system by t* = 0.8, and the material becomes more mixed over the remaining experiment time. In the water-GS experiment (Fig. 5d), one isolated lobe reaches the top of the slot at t* = 0.2, a timestep later than the other experiments. The material in this isolated central lobe becomes more mixed over time and is joined by a second lobe by t* = 0.6. At t* = 0.9, only one lobe is present at the top of the slot and is offset from the centre (the lobes of mixed material present in the previous timestep no longer reach as high in the slot and are replaced by downwelling unmixed material; Video Fig. S1 in Online Resource 2). More mixed material has begun to disperse across the slot length by the end of the water-GS experiment, but overall, the fluids at the top of the slot are not as mixed as in the other experiments.

**Fig. 6:**
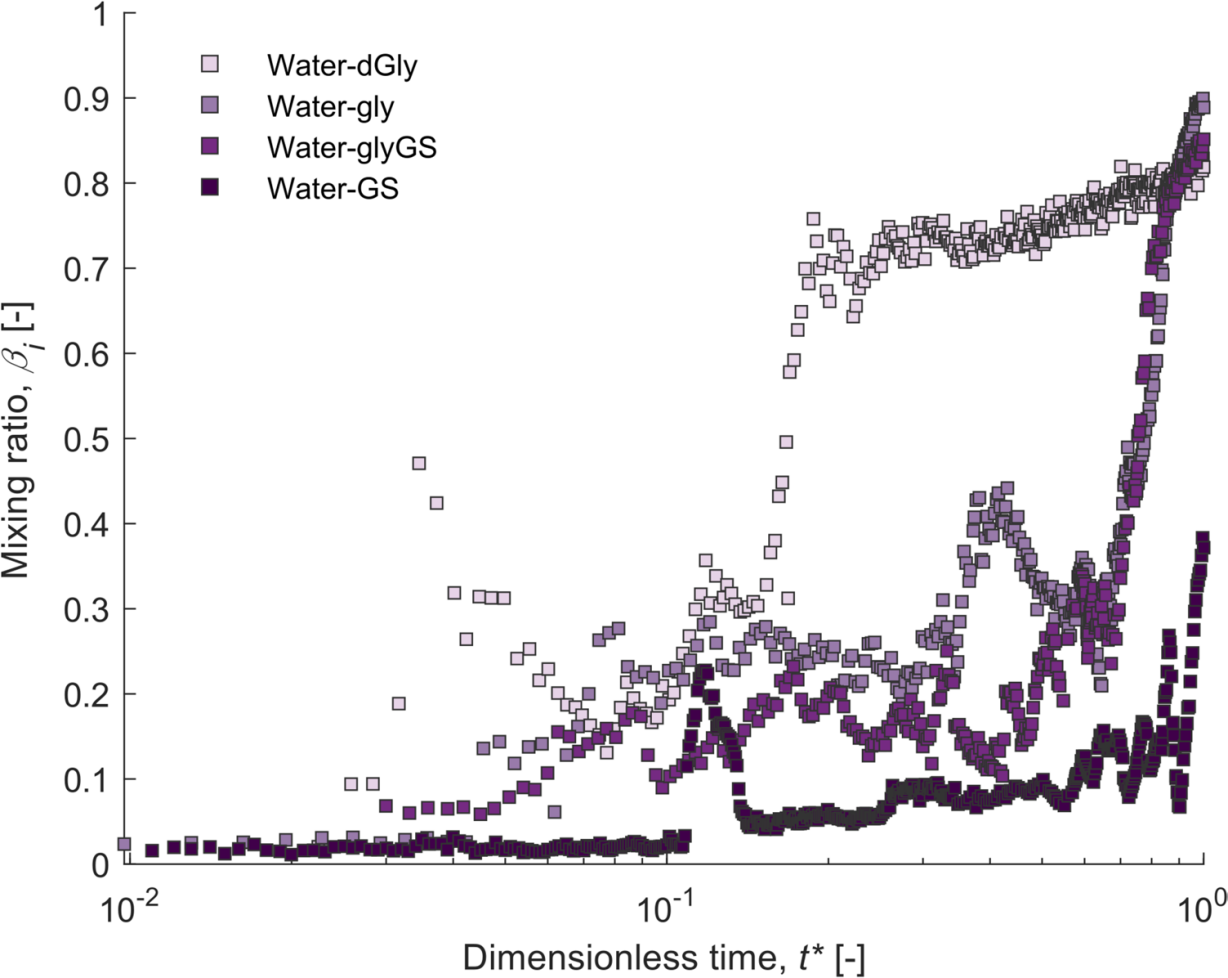
Plot of mixing ratio, *β*_*i*_,_*,*_ for the bulk material from a measurement profile at ~ 76 cm height in the slot (where 1 indicates fully mixed and 0 indicates completely unmixed), against dimensionless time, t*, for all upwelling water experiments. Downwards spikes in the *β*_*i*_ trend mean more unmixed material has reached the measurement profile, which lowers the value, before *β*_*i*_ recovers to the previous high as this material is mixed in. Water-dGly = lightest purple, water-gly = light purple, water-glyGS = dark purple, water-GS = darkest purple

We can observe the bulk mixing trend for all the water experiments by normalising *β*_sum_ to the maximum possible *β*_sum_ of an experiment to give a mixing ratio, *β*_*i*_ (Fig. 6) where a value closer to 1 indicates that there is more mixed material present. Downwards spikes in the *β*_*i*_ trend mean more unmixed material has reached the measurement profile, which lowers the value, before the material is incorporated and *β*_*i*_ recovers to the previous high as this material is mixed in. This is the case for water-dGly which shows a relatively rapid increase in mixing, with a few downwards spikes evident in the mixing ratio trend related to larger batches of less mixed material reaching the measurement profile. The less mixed material was subsequently incorporated, and the system returned to its previous high value, before continuing to further mix. The material in the measurement profile by t* = 0.2 is quite mixed for water d-Gly, after which there is a much slower increase of *β*_*i*_ until the reservoir is exhausted. Water-Gly, water-glyGS, and water-GS also show similar trends of downward spikes in the *β*_*i*_ trends but with a greater number of reductions compared to water-dGly. This continues until t* ≈ 0.7 for water-Gly and water-glyGS, after which the *β*_*i*_ rapidly increases to its final value. For water-GS, by the time the reservoir is exhausted, the *β*_*i*_ is still oscillating, and the final *β*_*i*_ is less than half that of the other fluid combinations, indicating that the material has still not completely mixed when there is no more upwelling fluid remaining.

**Fig. 7:**
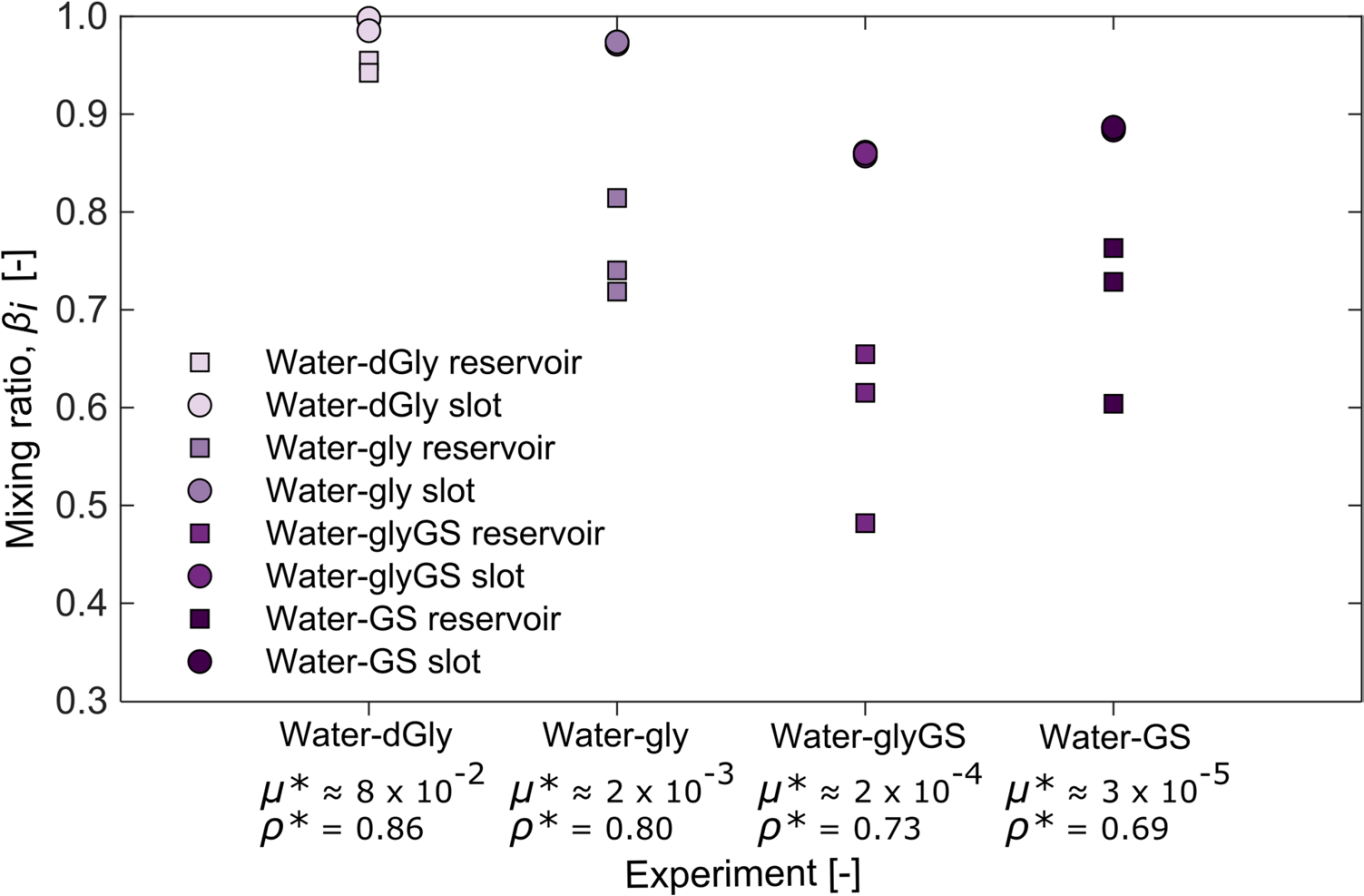
Plot of mixing ratio *β*_*i*_ for samples collected from the reservoir (square symbol) and the upper slot (circle symbol) at the end of each experiment where water was the upwelling fluid. Viscosity and density ratios, µ* and ρ*, respectively, are stated for each fluid pair. Symbol colour ranges from light to dark purple for decreasing ρ*

At the experiment end, we investigate the extent of mixing within different geometries by using fluid samples collected from both the upper reservoir (chamber geometry) and slot (dyke geometry; Fig. 1) to compare mixing ratios based on the sample densities at the end of the experiment (using the density as variable *x* in Eqs. (1) and (2), where *x*_*m*_ = ideal mixed fluid density, i.e. here, bulk density, *ρ*_*b*_). The densities of samples collected from the slot are more homogeneous than that of the reservoir, and the densities of the samples collected in the slot are much closer to that of the final bulk density (i.e. higher *β*_*i*_; Fig. 7).

## Discussion

### Dynamic controls on magma mixing

The findings of our analogue experiments indicate that flow is initially localised towards the centre of the geometry, and despite that the fluids are miscible, there is qualitative similarity between some of the initial flow patterns and those of immiscible fluids exchanging in a slot (Jones and Llewellin 2021). Mixing is focussed at the interface between the miscible fluids, and the extent of mixing gradually spreads laterally out across the breadth of the model dyke. Systems where the two endmember fluids have the most similar viscosity and density values (µ* and ρ* near to 1) mix quicker compared to systems where the endmembers differed more. In addition, at low viscosity ratios (i.e. when the two exchanging fluids differ substantially in viscosity), even for theoretically miscible fluids, the fluids behave in an immiscible manner for durations longer than the experiment timescale meaning the extent of mixing potential is limited over the exchange flow timescale of the system (e.g. W4_W-GS, Table 2; Fig. 5d; Fig. 6).

Using our tank design, we can also demonstrate how a chamber or dyke geometry affects mixing. The two reservoirs which are attached to the slot/dyke geometry comprise a chamber-like geometry. For all experiments, *β*_*i*_ in the slot is greater and closer to unity (i.e. perfectly mixed) than the reservoir (Fig. 7). This suggests that there is greater mixing in the slot, perhaps due to higher fluid velocities in the slot relative to the chamber (i.e. Bernoulli’s principle) and/or relatively high boundary effects of the wall relative to the volume of fluid, which may increase the shear or friction that the fluids experience (e.g. Chalk and Kavanagh 2024; Das and Mallik 2020; Taisne and Jaupart 2011). 

### Crystal exchange along magma interfaces

Crystal exchange between different magmas has been used as evidence of magma mixing in nature, particularly in the absence of macroscopic indicators of mixing such as deposit colour change or the presence of enclaves (e.g. Cooper et al. 2019). Crystal exchange occurs at the contact interface between magmas and broadly takes two forms. First, the physical exchange of crystals, which may be recorded as anomalous crystal populations (e.g. crystal size or composition), zoning, and disequilibrium crystal textures (e.g. Ubide et al. 2014). Second, the chemical diffusive exchange of elements in the crystal lattice, enriching and/or depleting certain mobile elements relative to the starting magma compositions (e.g. Humphreys et al. 2010).

Our experimental results provide additional insights on how crystal exchange is facilitated in natural systems. Our experiments show that a greater number of mixing lobes, and hence longer interface lengths and greater interface surface areas, generally occur when the fluid pairs have more similar physical properties (i.e. µ* and ρ* closer to 1), indicating that the interaction of similar magmas may initially provide more opportunities for crystal and diffusive exchange as crystals can cross through multiple areas of melt with differing composition (e.g. Cheng et al. 2020). However, chemical, diffusive exchange between the two endmember magmas would also occur (e.g. Zhang and Gan 2022). The efficiency of chemical diffusion processes depends on the surface area (e.g. Morgavi et al. 2013; Perugini and Poli 2004; Perugini et al. 2003), which indicates that the systems with the greatest interface surface areas (higher µ* and ρ*) would diffuse and thus chemically homogenise the melt most efficiently. This therefore provides a timescale constraint on recording disequilibrium within crystals.

When the initial endmembers are similar to one another (i.e. µ* and ρ* closer to 1) and the viscosities are relatively low (e.g. Exp. W1_W-dGly; Table 2), the mixing process within the dyke may be rapid and so efficient such that the timescale required to (diffusively) record disequilibrium within the crystal population is slower than the timescale of magma mixing. Thus, in these cases, the crystals are likely to simply record two melt compositions: the initial/parent endmember melt composition at their core and the final homogenised melt composition at their rim. Conversely, when the endmembers are more dissimilar (i.e. µ* and ρ* further from 1) and/or the initial endmember viscosities are relatively high (e.g. Exp. D1_dGly-Gly; Table 2), mixing is slow and inefficient, meaning chemical disequilibrium is maintained over longer time periods. This could provide adequate time for crystal growth and diffusion within crystals to be recorded in the population and thus be more likely to record progressive and complex magma mixing events (e.g. multiple element enrichment events being recorded in crystals; Bergantz et al. 2015; Ubide and Kamber 2018).

### Magma mixing at Kīlauea

Kīlauea is a shield volcano on the Island of Hawaiʻi where magma mixing from at least three distinct magma sources was identified during the 2018 eruption (Gansecki et al. 2019). Near-real time geochemical data were collected during the eruption and analysed for a suite of trace and major elements which all evolve over time as magma mixing takes place, with this evolution reflected in the changing behaviour of the eruption (Gansecki et al. 2019). The lavas became increasingly less viscous and more voluminous over time as the mixing system evolved from early phase 1 lavas that were relatively cool, highly differentiated basalts (enriched in incompatible elements such as Zr and Nb, and relatively low in compatible major elements CaO and MgO) to hotter phase 3 basalts that had lower incompatible elements and higher CaO and MgO.

The near-real time data from the 2018 Kīlauea eruption allows comparison between natural magma mixing processes and our experimental mixing data. However, to aid this comparison, some data filtering from the study of Gansecki et al. (2019) was undertaken. Data from Fissure 17 were filtered out, as this was where more explosive behaviour was observed and a distinct, highly evolved andesite lava erupted. This filtering should therefore minimise the effect introduced by this third endmember magma source, making the dataset more comparable with our two endmember analogue experiments. Furthermore, if the sample emplacement time was unknown, the corresponding geochemical data were excluded.

**Fig. 8:**
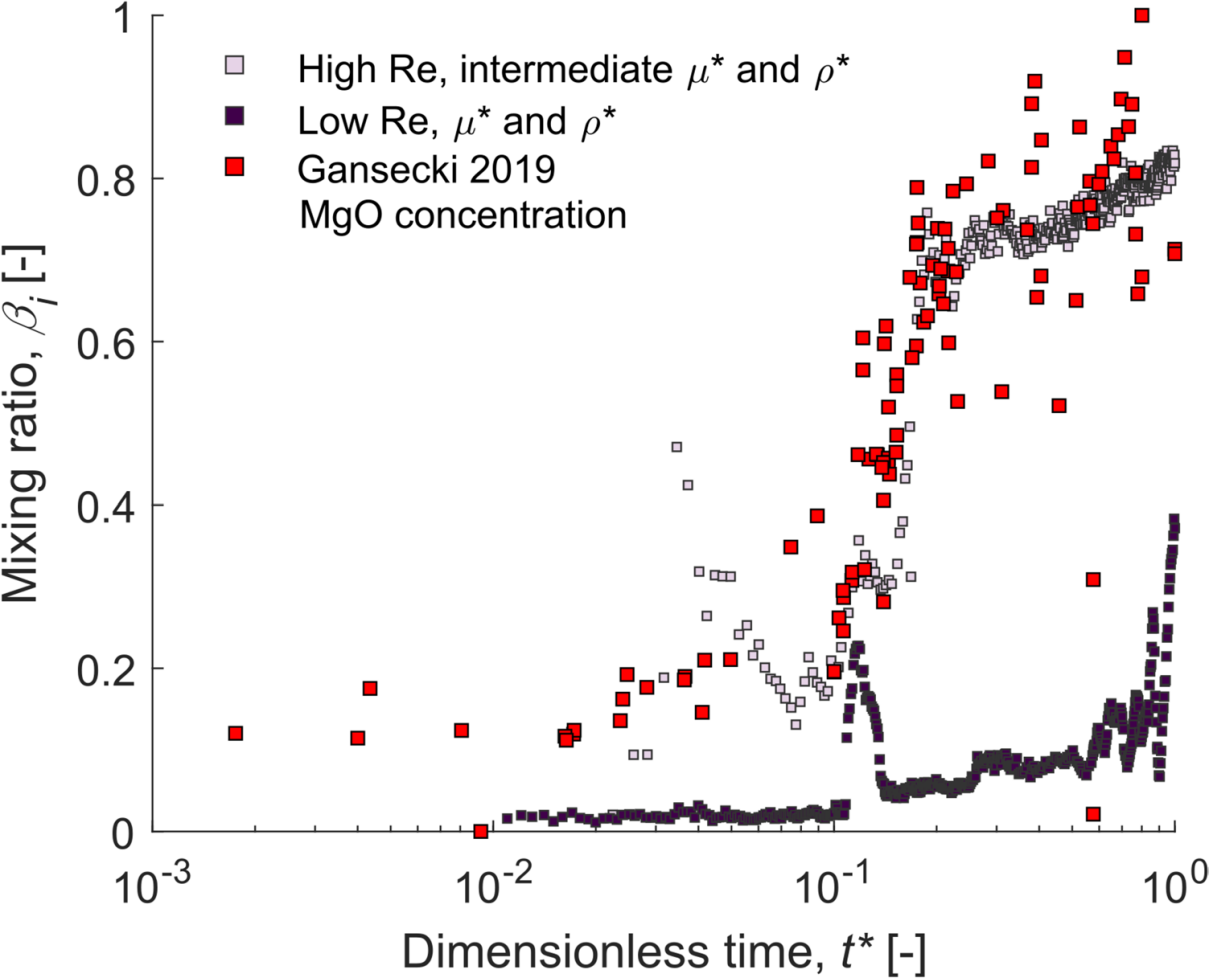
Plot of mixing ratio, *β*_*i*_, where 1 indicates fully mixed and 0 indicates not mixed, over dimensionless time, t*, for two experiments with varied physical properties (this work) and for normalised near-real time collected MgO (wt%) concentration data from the 2018 Kīlauea eruption (Gansecki et al. 2019). Light purple = high Reynolds (Re), intermediate viscosity and density ratio (µ* and ρ*) experiment; dark purple = low Re, µ* and ρ* experiment; red = normalised MgO data

As an illustrative example and to compare these natural data to our experiment, oxide concentration data were used. Specifically, here, we selected MgO concentration data as this increased from 4 to 5 wt% at the beginning of the eruption (Phase 1) up to 7–9 wt% by the eruption end (Phase 3), and MgO concentrations have been used in previous studies to identify magma endmembers and magma differentiation (usually MgO < 6.5 wt% indicates an evolved magma that has undergone extensive differentiation; Anderson et al. 2024; Gansecki et al. 2019). Equation (1) was used to normalise the data (equivalent MgO wt% values for variable *x*) and the time normalised by the eruption duration (120 days). Here, the geochemical data is compared to experimental data from water-dGly (Exp. W1_W-dGly; high Re, intermediate µ* and ρ*) and water-GS (Exp. W4_W-GS; low Re, µ* and ρ*) as they encompass a range of Reynolds numbers, viscosity ratios, and density ratios (Fig. 8; Table 3). It should be noted that the use of low, intermediate, and high to describe Re, µ*, and ρ* of the experiment system are relative to these experiments, not to natural ranges of these physical quantities. The other major and trace element concentrations (e.g., CaO, K_2_O, TiO_2_, Nb) also increased over time and showed similar trends to that of MgO (Fig. S3 in Online Resource 1).

**Table 3:** Fluid properties and dimensionless groups for the experimental and natural datasets in Fig. 8. The experimental datasets are reiterated from Table 2. For the Kīlauea 2018 data, here we use sample 3227 (Phase 1 early lava) and sample 3298 (Phase 3 lava) to represent the endmember fluids (respectively, the earliest and latest erupted samples given within Table S1 of Gansecki et al. 2019). From the aforementioned table, the viscosity (*µ*) values are used here, and the densities (*ρ*) were calculated by inputting representative glass compositions into DensityX (Iacovino and Till 2019). The Reynolds number (Re) for Kīlauea 2018 was calculated using Re = *ρ*_*d*_*Q/Dµ*_*d*_ where *Q* = 100 m^3^ s^−1^ (approximate effusion rate during phase 2; Dietterich et al. (2021)) and *D* = 1 m (assumed)

Dataset	Upwelling fluid	Downwelling fluid	*ρ*_*u*_	*ρ*_*d*_	*µ*_*u*_	*µ*_*d*_	ρ*	µ*	Re
-	-	-	kg m^−3^	kg m^−3^	Pa s	Pa s	-	-	-
W1_W-dGly	Water	61 wt% Gly	999	1159	9.3 × 10^−4^	1.2 × 10^−2^	8.6 × 10^−1^	7.6 × 10^−2^	5.7 × 10^1^
W4_W-GS	Water	100 wt% GS	999	1440	1.0 × 10^−3^	4.0 × 10^1^	6.9 × 10^−1^	2.6 × 10^−5^	1.9 × 10^−4^
Kīlauea 2018	Phase 3 lava	Phase 1 early lava	2716	2735	1.2 × 10^3^	6.7 × 10^3^	9.9 × 10^−1^	1.7 × 10^−1^	4.1 × 10^1^

Generally, the MgO concentration mixing ratio trend (red datapoints in Fig. 8) matches well with that of the water-dGly dataset (light purple datapoints in Fig. 8), and the calculated Re, µ*, and ρ* values for the Kīlauea 2018 dataset are within an order of magnitude to those in our water-dGly experiment (Table 3). In comparison, the mixing ratio trend of the water-GS experiment (dark purple datapoints in Fig. 8) differs greatly to the MgO concentration mixing ratio trend, and Re and µ* are 4–5 orders of magnitude lower than the Kīlauea values (Table 3). This indicates that the behaviour of the natural mixing system, where the magma endmembers are both basaltic and of similar viscosity (Table 3), may have been similar to that seen during our water-dGly experiment.

Although our experiment setup simplifies some of the complexity found in the natural system, our experimental data can still reproduce similar mixing trends to real geochemical datasets. Further development of this method could assist in future near-real time geochemical monitoring efforts. Factors that could additionally promote or hinder mixing in nature and need to be considered include localised temperature perturbations where hotter areas (i.e. lower viscosity) may mix more efficiently than cooler areas, multiphase magmas (crystals and/or bubbles in melt) which exhibit non-Newtonian rheology (Vasseur et al. 2023), and the surface roughness of the dyke walls where increased roughness may enhance mixing (e.g. Johnson et al. 2006).

### Exchange flow in volcanic conduits

**Fig. 9:**
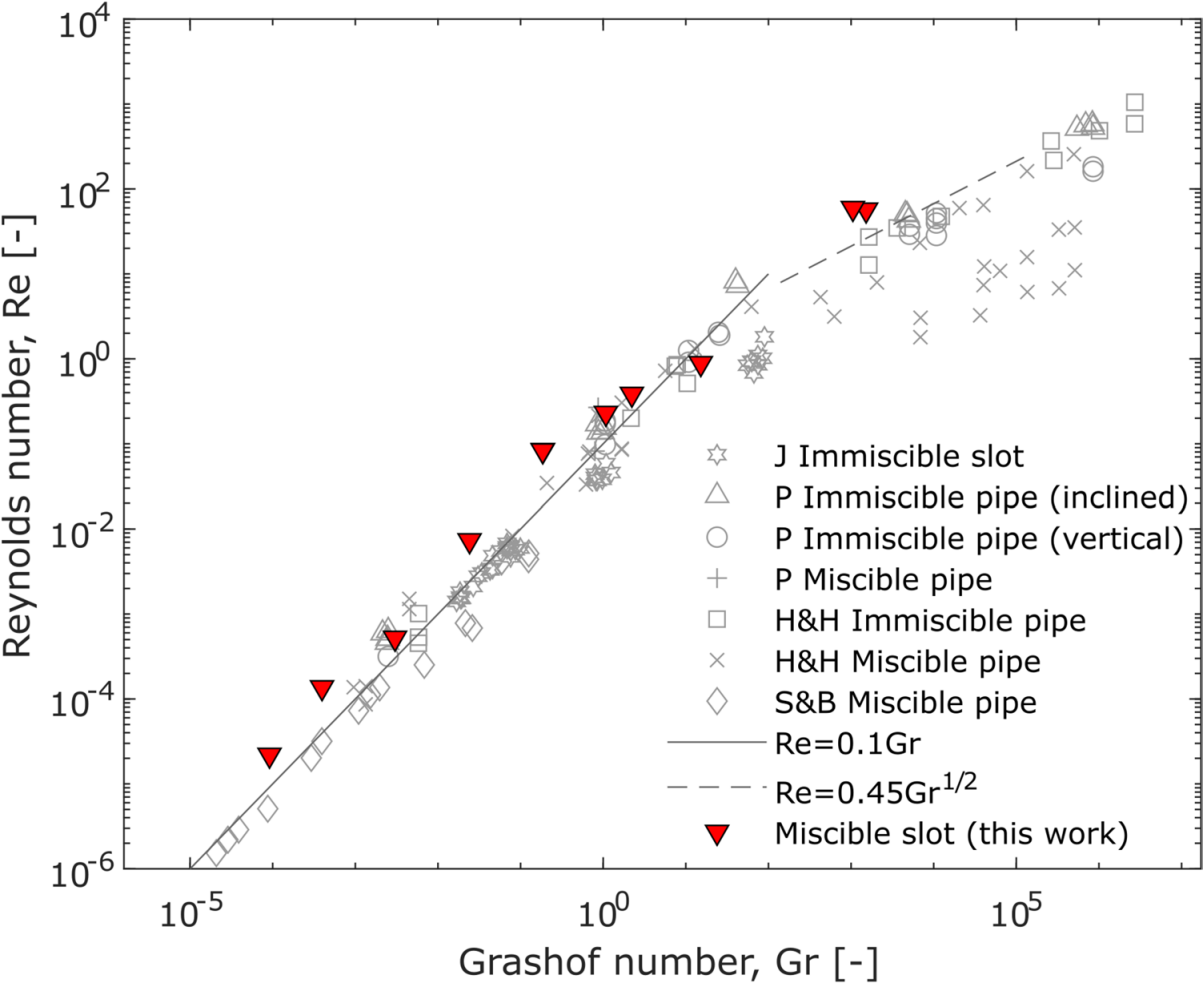
The linear relationship between the Reynolds number (Re) and Grashof number (Gr) for experimental data in this paper. Results from Palma et al. (2011; [P]) and data collated therein (Huppert and Hallworth (2007; [H&H]) Stevenson and Blake (1998; [S&B]), and Jones (2018; [J])) are shown for comparison. Red triangle symbols are data from this paper. Grey outline symbols are data from previous work. Miscible = miscible fluid pairs, immiscible = immiscible fluid pairs, slot = slot geometry tank, pipe = pipe geometry tank. Modified from Palma et al. (2011)

Our experiments can also inform on the dynamics of persistently active basaltic systems where a convective exchange flow occurs between fresh upwelling, volatile-rich magma and downwelling, dense outgassed magma (e.g. Kazahaya et al. 1994; Palma et al. 2011; Qin et al. 2021; Stevenson and Blake 1998). Previous experimental studies have investigated this process using cylindrical pipe-like geometries as analogues for the volcanic conduit (e.g. Beckett et al. 2011; Huppert and Hallworth 2007; Palma et al. 2011; Stevenson and Blake 1998) and typically use the Reynolds (Re) number and Grashof (Gr) number, as dimensionless groups to quantify the flow relationships (Fig. 9).

The Grashof number is a dimensionless group that represents systems of natural convection through the ratio of viscous to buoyancy forces which governs exchange flow (e.g. Beckett et al. 2011; Jones and Llewellin 2021). It represents a buoyancy-driven equivalent of the Reynolds number and can be expressed as:

8 $$Gr= \frac{g^{\prime}{D}^{3}{\rho }_{d}^{2}}{{\mu }_{d}^{2}}$$

where *g’* = *g* cos*θ ∆ρ/ρ*_*d*_ (Palma et al. 2011), *g* is the standard acceleration due to gravity (9.81 m s^−2^), *θ* is the angle of inclination of the conduit (0° is vertical, 90° is horizontal), and *Δρ* is the density difference between the two fluids. The crack/slot thickness, *D* is the characteristic length scale as *D* is the major factor controlling volumetric flux in fissures and dykes and their flow behaviour (e.g. Delaney and Pollard 1982).

Our experimental results overlap with previous works (Fig. 9) and demonstrate that Re is a function of Gr for our mixing experiments in a slot geometry. Furthermore, our quantitative Re-Gr relationship is also in agreement with previous experiments performed using pipe geometry with immiscible and miscible fluids. Specifically, for Gr < ~ 10^2^, the relationship is Re = 0.1 Gr. When Gr > ~ 10^2^, Palma et al. (2011) identified a deviation from this relationship and found that Re = 0.45 Gr^1/2^. In this study, we only have two datapoints in this high Gr regime, and, although in agreement with the previous pipe experiments, a strong confirmation cannot be made. However, the agreement in the Re-Gr relationship shown here (Fig. 9) supports extrapolation of relationships identified in the pipe case to a dyke-like geometry.

## Conclusions

We present an analogue model for mixing of miscible magmas within a dyke-like geometry. Our experiments show that flow is initially localised towards the centre of the system, and that mixing occurs at the interface between the different fluids and spreads out laterally across the analogue dyke length (i.e. its strike) over time. For experiments where there is common starting fluid, mixing occurs more quickly and is more efficient with density and viscosity ratios closer to 1. However, at viscosity ratios further away from 1 (µ* << 1 or µ* >> 1; e.g. W4_W-GS, Table 2, µ* = 2.6 × 10^−5^), i.e. when the two exchanging fluids differ substantially in viscosity, even for theoretically miscible fluids, the fluids will not fully mix over the exchange flow timescale of the system.

Our results also show the geometry of a magma system likely affects mixing with dykes enabling more mixing relative to more chamber-like magma systems, and that the dynamics of convective exchange flow in persistently active basaltic systems identified in pipe-like geometries can also be extrapolated to dyke-like geometries. Melt endmembers within a dyke, with low viscosities and more similar properties (i.e. µ* and ρ* closer to 1), are expected to undergo fast and efficient mixing, meaning there may be insufficient time for the crystals suspended in the magma to record the complexities of the mixing process. In contrast, high-viscosity melt endmembers within a dyke would mix at much longer timescales, and therefore their crystal cargo may contain a more complete chemical record of the mixing process. Comparable mixing ratio trends shown by our experimental results are also seen by near-real time geochemical datasets of magma mixing in a dyke-fed eruption in nature with similar Reynolds number, viscosity ratio, and density ratio values (within an order of magnitude). This suggests the experiments are dynamically similar to that of natural examples and, with further development, they could be useful in future hazard monitoring tools.

## Supplementary Information

Below is the link to the electronic supplementary material.

Supplementary file1 (DOCX 2.02 MB)

Supplementary file2 (MP4 108 MB)

## Data Availability

Data are included within the main article and its supplementary information files. The raw material and codes used to process the images have been submitted to the National Geoscience Data Centre (NGDC). 10.5285/b0634651-410d-4531-8100-e1d4e2873d9e

## Code availability

Matlab codes used to process the images have been submitted to the National Geoscience Data Centre (NGDC). 10.5285/b0634651-410d-4531-8100-e1d4e2873d9e
