# Supplementary material for: Analogue experiments to investigate magma mixing within dykes
Source: Bull Volcanol. 2025 Mar 28;87(4):29. doi: 10.1007/s00445-025-01809-0 (PMC11976769; doi:10.1007/s00445-025-01809-0)
Supplement: Supplementary file 1 — Supplementary file1 (DOCX 2.02 MB) [file 445_2025_1809_MOESM1_ESM.docx]

# Supplementary data

**Online Resource 1: Supplementary figures showing pixel value and density relationship, image processing, and normalised timeseries geochemical data from the 2018 Kīlauea eruption**

| **Fig. S1** Plot showing linear relationship between average pixel value of the slot area and changing density as different proportions of two different density fluids are mixed (denser fluid dyed). Error bars are smaller than the data points, ± 0.003, the maximum range in the average pixel value of the slot for ten photos taken under camera and lighting conditions described in Section ‘Experimental procedure’ |
| --- |
| 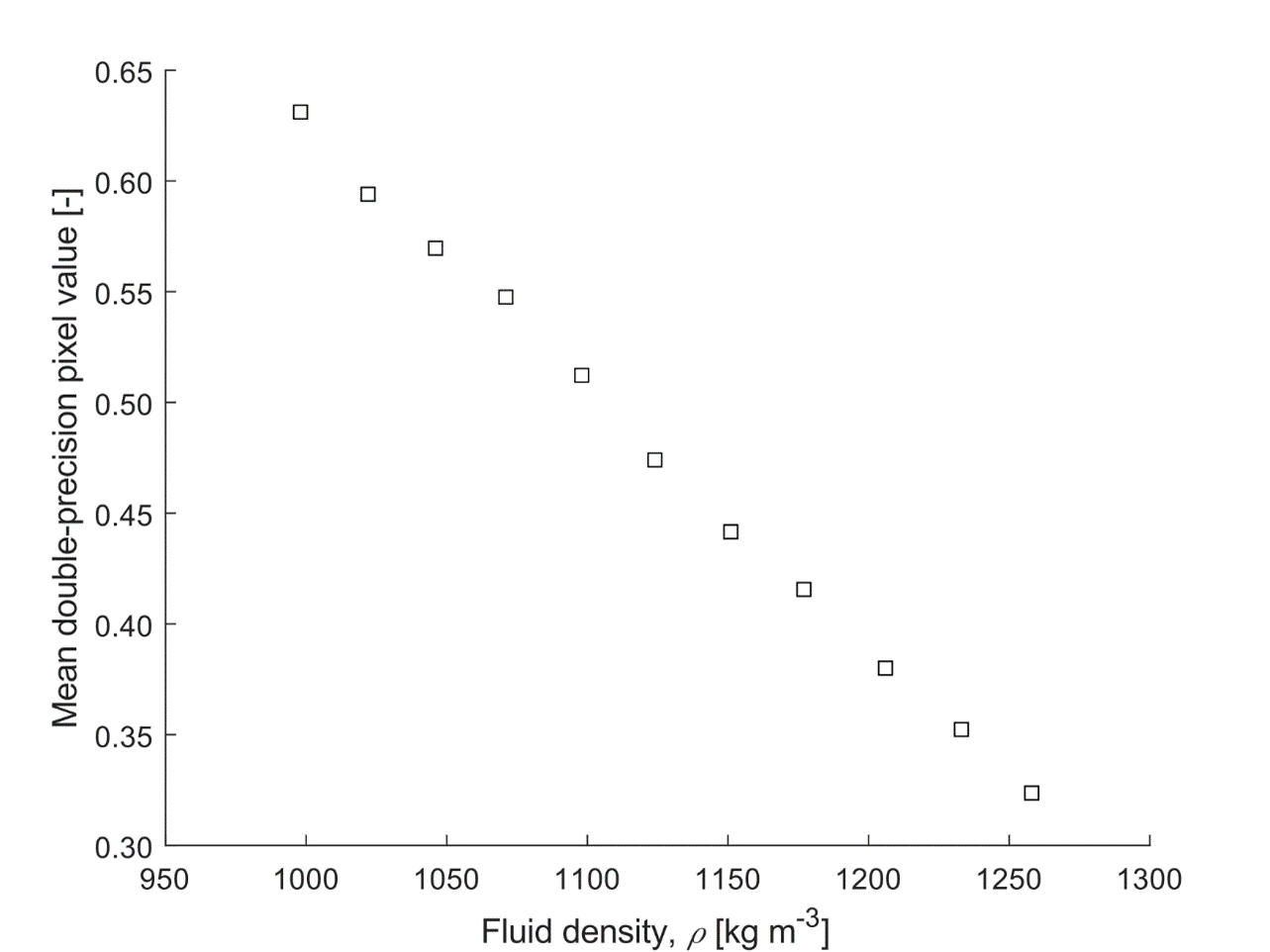 |

| **Fig. S2** Example of processed image showing conversion from **(a)** the original image to the **(b)** colourmap determined by the pixel value. This represents the fluid density. The blue end of the spectrum represents denser fluid and red represents less dense fluid |
| --- |
| 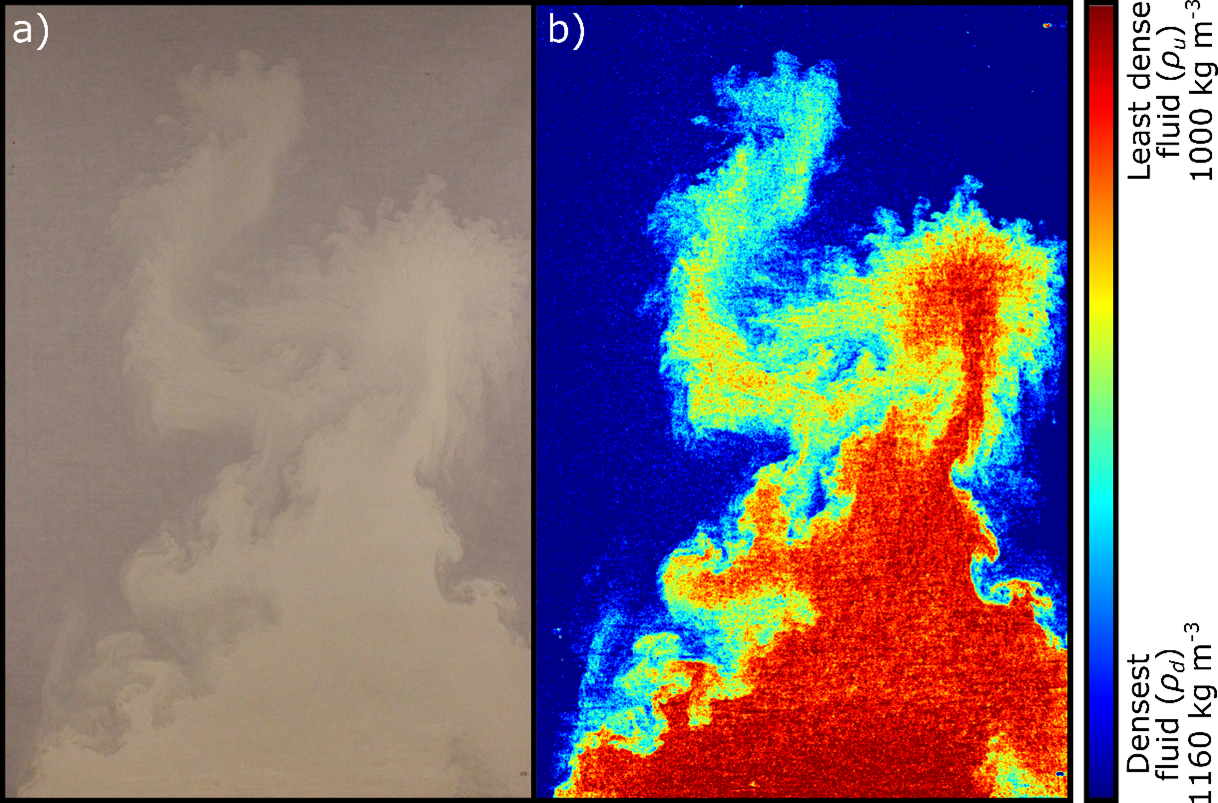 |

| **Fig. S3** Plot of normalised real-time collected major and trace element concentration data from the 2018 Kīlauea eruption (Gansecki et al. 2019) over dimensionless time, *t**. Major elements are filled symbols: **(a)** CaO, **(b)** K_2_O, **(c)** TiO_2_ and **(d)** Na_2_O. Trace elements are black outline symbols: **(e)** Nb, **(f)** Rb, **(g)** Sr, **(h)** Y, and **(i)** Zr |
| --- |
| 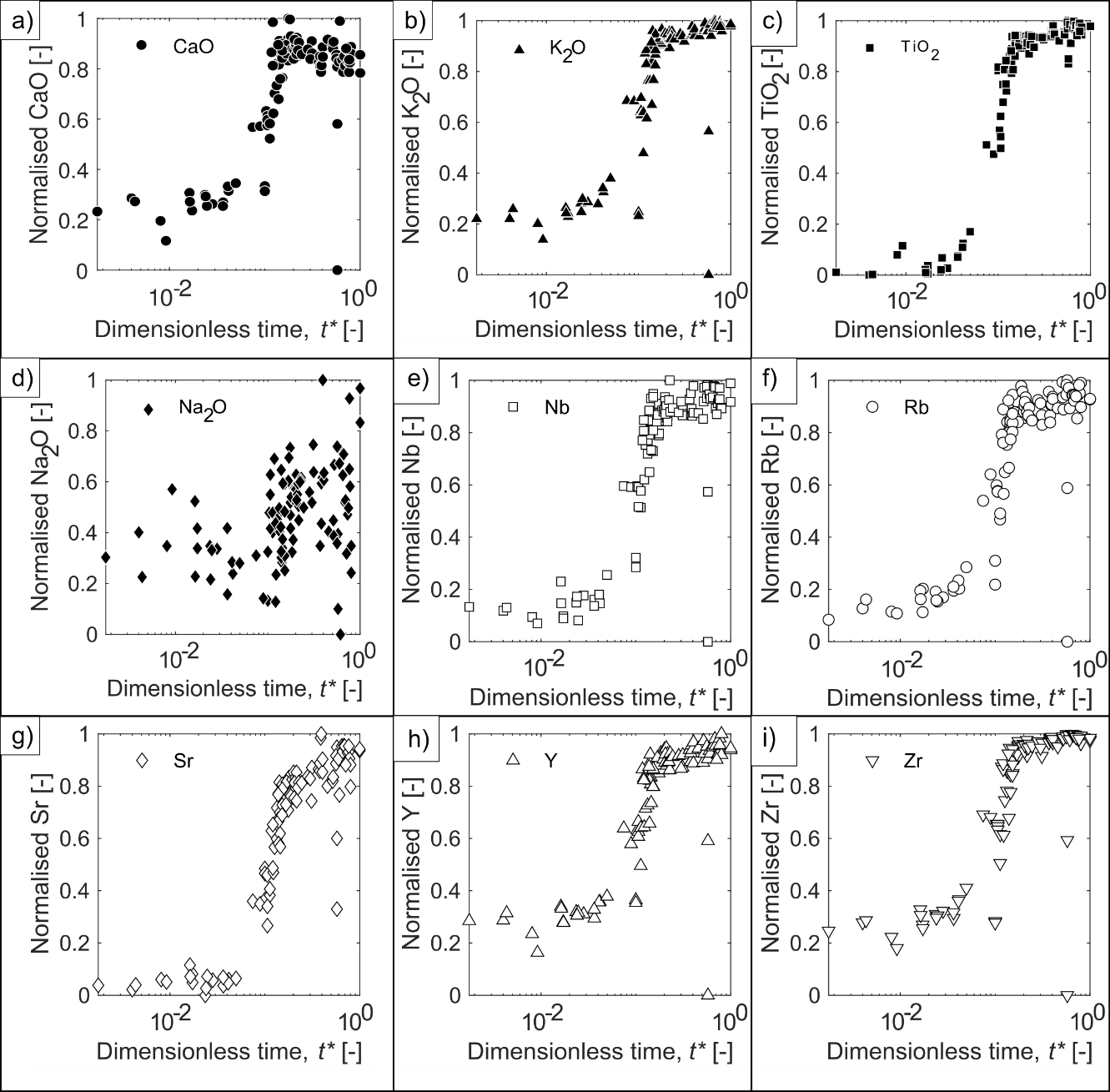 |

**Online Resource 2: Videos of fluid pair experiments**

**Video Figure S1** Fluid interaction experiment videos where a low density upwelling fluid progressively mixes with a dense downwelling fluid in a slot. In all videos the original (left) images are shown and processed (right) images show low density (red), high density (blue) and progressive mixing at the experiment run time (in seconds). Scale for all images ≈ 50 x 80 cm (Length x height). The Reynolds number, Re, viscosity ratio *µ** and density ratio *ρ** are shown for each fluid pair. **(a)** Exp. W1_W-dGly, **(b)** Exp. W2_W-Gly, **(c)** Exp. W3_W-Gly-GS, **(d)** Exp. W4_W-GS, **(e)** Exp. D1_dGly-Gly, **(f)** Exp. G1_Gly-dGS, **(g)** Exp. G2_Gly-GS
